# GGT‐Normal Cholestasis in an Older Child: A Suspected Case of Benign Recurrent Intrahepatic Cholestasis From Syria

**DOI:** 10.1002/ccr3.72950

**Published:** 2026-06-12

**Authors:** Ahmad Hosiian, Balsam Aldali, Jihan Jendi, Mostafa Hassan, Maria Naamah, Ali Amhanna, Maya Moustafa

**Affiliations:** ^1^ Department of Infectious Diseases Tartous National Children's Hospital Tartous Syria; ^2^ Department of Neurosurgery Tartous University Tartous Syria; ^3^ Department of Pediatric Gastroenterology Latakia University Latakia Syria

**Keywords:** benign recurrent intrahepatic cholestasis (BRIC), case report, cholestasis, normal gamma‐glutamyl transferase, pediatric hepatology

## Abstract

Cholestasis beyond infancy is uncommon and presents a diagnostic challenge. We report an 11‐year‐old girl presenting with severe pruritus followed by jaundice and a cholestatic biochemical profile with normal gamma‐glutamyl transferase levels. Extensive evaluation excluded infectious, autoimmune, metabolic, and obstructive causes. Liver biopsy demonstrated bland cholestasis with preserved hepatic architecture. The patient showed complete clinical and biochemical recovery following supportive therapy with ursodeoxycholic acid. Although this represents a first documented episode, the findings are most consistent with a benign recurrent intrahepatic cholestasis–like phenotype. This case highlights the importance of considering inherited cholestatic disorders in older children with unexplained GGT‐normal cholestasis.

## Key Clinical Message

GGT‐normal cholestasis with severe pruritus in older children should raise suspicion for inherited bile acid transport disorders. Even after a first episode, a BRIC‐like phenotype may be considered once common causes are excluded, highlighting the importance of long‐term follow‐up, especially in resource‐limited settings without access to genetic testing.

## 1 Introduction

Cholestasis in older children is uncommon and often presents a diagnostic challenge due to its wide etiologic spectrum and variable clinical manifestations [1]. A structured evaluation is essential to distinguish self‐limited functional disturbances from progressive hepatobiliary disease. Among the rare intrahepatic causes, benign recurrent intrahepatic cholestasis (BRIC) remains underrecognized, particularly in regions with limited access to molecular testing [2]. We report a cholestatic episode in an 11‐year‐old girl that, after comprehensive exclusion of common etiologies, was most consistent with BRIC, representing, to our knowledge, the first documented case from Syria.

## 2 Case History/Examinations

An 11‐year‐old Syrian girl presented with a two‐week history of severe generalized pruritus, followed by progressive jaundice. Associated symptoms included anorexia, abdominal pain, low‐grade fever, and dark‐colored urine. There was no history of pale stools. She had received antihistamines before presentation without symptomatic relief.

On examination, the patient appeared pale. Her vital signs were stable, with a heart rate of 80 beats/min, respiratory rate of 16 breaths/min, blood pressure of 110/70 mmHg, body temperature of 37°C, and oxygen saturation of 98% on room air. Cardiovascular examination revealed a regular rhythm with no audible murmurs, and pulmonary auscultation was clear bilaterally. Abdominal examination showed a soft, non‐tender abdomen with no organomegaly. Neurological examination was unremarkable.

## 3 Differential Diagnosis, Investigations, and Treatment

Initial laboratory investigations demonstrated a cholestatic pattern of liver injury: aspartate aminotransferase (AST) 90 U/L, alanine aminotransferase (ALT) 70 U/L, total bilirubin 16 mg/dL, and direct bilirubin 11.4 mg/dL. Alkaline phosphatase (ALP) was elevated at 655 IU/L, while gamma‐glutamyl transferase (GGT) was within the normal range at 11.4 IU/L. Coagulation parameters were normal (INR 1.2). A summary of laboratory findings is provided in Table 1.

**TABLE 1:** Laboratory findings from admission through the final follow‐up at six months.

Laboratory tests	Initial tests	One week later	After taking UDCA	One month later	Three months later	Six months later
Aspartate aminotransferase (AST) (U/L)	90	44	42	—	—	—
Alkaline transaminase (ALT) (U/L)	70	35	30	—	—	—
Total bilirubin (mg/dL)	9.3	16	4.9	1.6	0.3	0.5
Direct bilirubin (mg/dL)	—	11.4	4.2	1.16	—	0.1
Alkaline phosphatase (U/L)	655	—	328	—	—	—
Leukocytes (cells/μL)	5500	—	—	—	—	—
Hemoglobin (g/dL)	13	—	—	—	—	—
Plateletes (μL)	351,000	—	—	—	—	—
Urea (mg/dL)	25	—	—	—	—	—
Creatinine (mg/dL)	0.9	—	—	—	—	—
Prothrombin time (PT) (s)	15.9	—	13.5	—	—	—
Patial thromboplastin time (PTT) (s)	31	—	30	—	—	—
International normalized ratio	1.2	—	1	—	—	—
Gamma‐glutamyl transferase (U/L)	11.4	—	15	12	—	10.1
Ceruloplasmin (mg/dL)	41	—	—	—	—	—
24 h urine copper (μg)	22.8	—	—	—	—	—
Anti‐HAV‐IgG	0.2 (N)					
Anti‐HAV IgM	0.1 (N)					
HbsAB IU/mL	0.3 (N)					
HC AB	Non‐reactive (N)					
ANA	(N)					
ASMA	(N)					
AMA	(N)					
Anti‐LKM AB	(N)					

Abdominal ultrasonography showed normal liver echotexture and biliary anatomy, with no evidence of extrahepatic biliary obstruction. Viral hepatitis serologies, including hepatitis A, B, and C, were negative. Autoimmune evaluation, including antinuclear antibody, anti–smooth muscle antibody, anti–liver–kidney microsomal antibody, and antimitochondrial antibody, was negative. Ceruloplasmin level was normal, and there was no biochemical evidence of Wilson disease. Urinalysis was unremarkable.

**FIGURE 1:**
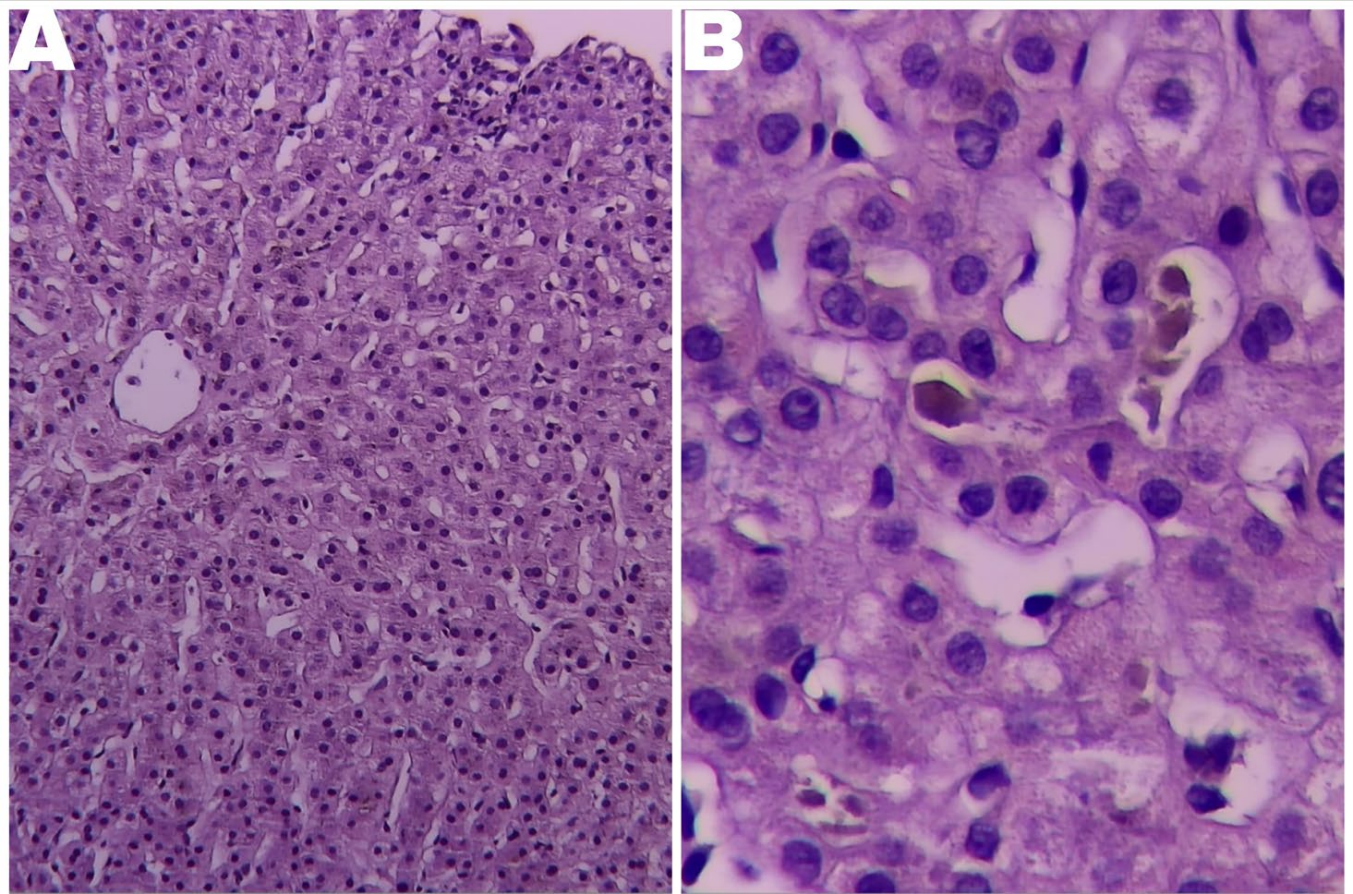
Histopathological features of centrilobular cholestasis. (A) Low‐power view (H&E stain, ×100) showing preserved hepatic architecture with centrilobular accumulation of bile within hepatocytes surrounding the central vein, consistent with hepatocellular cholestasis. No significant inflammation or fibrosis is identified. (B) High‐power view (H&E stain, ×400) demonstrating occasional canalicular bile plugs.

Liver biopsy revealed preserved hepatic architecture with prominent hepatocellular and canalicular cholestasis. Inflammatory infiltrates were minimal, and there was no evidence of interface hepatitis, bile duct injury, ductopenia, or fibrosis. These findings were consistent with a bland cholestatic process (Figure 1).

Following exclusion of obstructive and secondary causes of cholestasis, the patient was started on supportive therapy. Ursodeoxycholic acid (UDCA) was initiated at a dose of 15 mg/kg/day in two divided doses. Symptomatic treatment for pruritus included oral antihistamines and skin emollients. Adequate hydration and nutritional support were maintained, with supplementation of fat‐soluble vitamins (A, D, E, and K). Gradual clinical improvement in pruritus was observed over the following weeks, followed by progressive biochemical recovery.

A detailed history revealed no exposure to medications, herbal remedies, or known hepatotoxins. There was no history of recent travel, prior similar episodes, or family history of recurrent jaundice or chronic liver disease.

## 4 Conclusion and Results (Outcome and Follow‐Up)

At a one‐week follow‐up, liver enzymes had significantly improved (AST 44 U/L, ALT 35 U/L), and symptoms markedly improved, although jaundice persisted. UDCA therapy was then initiated (Table 1).

At one‐month follow‐up, laboratory tests showed continued improvement: AST 42 U/L, ALT 30 U/L, ALP 328 IU/L, total bilirubin 4.9 mg/dL, direct bilirubin 4.2 mg/dL, with persistently normal GGT levels. At the three‐month follow‐up, total bilirubin had decreased to 0.5 mg/dL and subsequently normalized to 0.3 mg/dL by six months, after which the UDCA dose was gradually tapered.

## 5 Discussion

Pediatric cholestasis represents a heterogeneous group of disorders characterized by impaired bile formation or flow, typically manifesting as direct hyperbilirubinemia with a cholestatic biochemical profile [1]. A structured diagnostic approach is essential to distinguish self‐limited functional disorders from progressive hepatobiliary disease [1, 2].

In this case, an 11‐year‐old girl presented with subacute cholestasis marked by intense pruritus preceding jaundice, disproportionately elevated cholestatic markers, normal γ‐glutamyl transferase (GGT), and only mild aminotransferase elevation. Imaging studies excluded extrahepatic biliary obstruction, and liver biopsy demonstrated a cholestatic pattern of injury with minimal inflammation and preserved hepatic architecture. Following a systematic diagnostic evaluation, common infectious, autoimmune, metabolic, and obstructive causes of pediatric cholestasis were excluded [2, 3].

The combination of severe pruritus, normal GGT, absence of structural biliary disease, and benign histological findings prompted consideration of inherited disorders of bile acid transport. Progressive familial intrahepatic cholestasis (PFIC) was considered unlikely given the lack of progressive disease, normal GGT levels, absence of fibrosis or ductal abnormalities on biopsy, and complete biochemical resolution [4]. Similarly, syndromic causes such as Alagille syndrome were excluded based on clinical features [5].

Taken together, this presentation is most consistent with a suspected benign recurrent intrahepatic cholestasis (BRIC)–like phenotype. Although BRIC is classically defined by recurrent episodes, the current case demonstrates several hallmark features, including predominant cholestasis with preserved liver architecture and symptomatic and biochemical improvement following ursodeoxycholic acid (UDCA) therapy [4, 6]. Although the role of UDCA in BRIC is not clearly established, it is commonly used for symptomatic relief and may contribute to biochemical improvement. Given that this represents the patient's first documented episode, a definitive diagnosis cannot yet be established, and long‐term follow‐up is warranted to assess for recurrence.

BRIC and PFIC are now recognized as part of a phenotypic continuum related to defects in hepatocellular bile transport proteins, most commonly involving ATP8B1, ABCB11 (BSEP), or ABCB4 (MDR3) [6, 7]. Genetic testing was not available in this resource‐limited setting; however, molecular evaluation remains important for diagnostic confirmation, prognostication, and counseling, and is being pursued when feasible [7]. Nevertheless, the absence of molecular confirmation does not preclude a clinical diagnosis in settings where genetic testing is unavailable.

It is also important to distinguish this presentation from acute hepatitis of unknown etiology recently described in children, which is characterized by marked aminotransferase elevation and hepatocellular injury [8, 9]. In contrast, our patient exhibited a clearly cholestatic pattern with minimal inflammatory changes and rapid recovery, making this alternative diagnosis unlikely.

This case highlights the diagnostic challenge posed by isolated GGT‐normal cholestasis in older children and underscores the importance of considering BRIC in the differential diagnosis, even after a first episode, particularly in resource‐limited settings [4, 6]. To our knowledge, this represents the first reported suspected case of BRIC from Syria, contributing to regional awareness of rare pediatric cholestatic disorders. Long‐term follow‐up is planned to monitor for recurrence and to reconsider genetic testing should further episodes occur.

An important limitation in the present case is that BRIC is classically defined by recurrent episodes of cholestasis separated by symptom‐free intervals. Because this represents the patient's first documented episode, the diagnosis should be considered provisional and based on a BRIC‐like phenotype rather than confirmed BRIC. Long‐term follow‐up is therefore essential. Recurrence of cholestatic episodes over time would strongly support BRIC, whereas the development of persistent cholestasis, growth impairment, fibrosis, or progressive liver dysfunction would raise concern for PFIC, which lies on the same clinical spectrum. Periodic clinical and biochemical monitoring is planned, with reconsideration of genetic testing should recurrence occur. This longitudinal approach is particularly important in resource‐limited settings where molecular confirmation is not immediately available.

## Author Contributions

**Ahmad Hosiian:** conceptualization, investigation, project administration, supervision, visualization, writing – original draft, writing – review and editing. **Balsam Aldali:** investigation, writing – original draft, writing – review and editing. **Jihan Jendi:** investigation, writing – original draft, writing – review and editing. **Mostafa Hassan:** data curation, methodology, resources, software, writing – original draft, writing – review and editing. **Maria Naamah:** data curation, investigation. **Ali Amhanna:** writing – review and editing. **Maya Moustafa:** writing – review and editing.

## Funding

The authors have nothing to report.

## Ethics Statement

The authors have nothing to report.

## Consent

Written informed consent was obtained from the parents of the patient to publish this report in accordance with the journal's patient consent policy.

## Conflicts of Interest

The authors declare no conflicts of interest.

## Data Availability

The data underpinning the findings of this study are available from the corresponding author upon reasonable request. Public availability of the dataset is limited due to privacy and ethical concerns.
